# A new decellularized tendon scaffold for rotator cuff tears – evaluation in rabbits

**DOI:** 10.1186/s12891-020-03680-w

**Published:** 2020-10-17

**Authors:** Alex de Lima Santos, Camila Gonzaga da Silva, Leticia Siqueira de Sá Barreto, Katia Ramos Moreira Leite, Marcel Jun Sugawara Tamaoki, Lydia Massako Ferreira, Fernando Gonçalves de Almeida, Flavio Faloppa

**Affiliations:** 1 Department of Orthopedic and Traumatology, EPM - Federal University from São Paulo, 715 Napoleão de Barros, São Paulo, SP 04038-000 Brazil; 2 Discipline of Urology, Department of Surgery, EPM - Federal University of Sao Paulo, São Paulo, Brazil; 3 Department of Surgery, University of Sao Paulo, São Paulo, Brazil; 4 Discipline of Plastic Surgery, Department of Surgery, EPM - Federal University of Sao Paulo, São Paulo, Brazil

**Keywords:** Scaffold, Tissue engineering, Extracellular matrix, Decellularization, Tendon

## Abstract

**Background:**

Scaffolds have considerably advanced in recent years. In orthopaedic surgery, scaffolds have been used as grafts in procedures involving tendon and ligament reconstruction. This paper aimed to produce and evaluate decellularized tendon scaffolds (DTSs) from biomechanical, microscopic, macroscopic and in vivo perspectives.

**Methods:**

Bilateral gastrocnemius muscle tendons from 18 adult New Zealand rabbits were collected. Of these 36 tendons, 11 were used as controls (Group A - control), and 25 were used in the decellularization protocol (Group B - DTS). The groups were subjected to histological, biomechanical and macroscopic analyses, and Group B - DTS was subjected to an additional in vivo evaluation. In the decellularization protocol, we used a combination of aprotinin, ethylenediamine tetraacetic acid (EDTA), sodium dodecyl sulfate (SDS) and t-octyl-phenoxypolyethoxyethanol (Triton X-100) for six days. During this period, the scaffolds were kept at room temperature on an orbital shaker with constant motion.

**Results:**

The DTSs showed an increased cross-sectional area and inter-fascicular distance and no change in parallelism or matrix organization. The nuclear material was not organized in the DTSs as it was in the control. In the biomechanical analysis, no significant differences were found between the groups after analysing the ultimate tensile load, stiffness, and elongation at the ultimate tensile load. During the in vivo evaluation, mononuclear cell infiltration was noted.

**Conclusions:**

The evaluated decellularization protocol generated a tendon scaffold, maintained the most important biomechanical characteristics and permitted cell infiltration.

## Background

Rotator cuff tears are a common cause of shoulder disability and are normally associated with weakness, loss of motion, debilitating pain and a reduced capacity for daily activities [1]. Treatment ranges from “wait-and-see” approaches to surgery. Typically, the treatment is selected according to the patient characteristics, tear size and symptoms [2, 3]. In patients with large and massive rotator cuff tears submitted for surgical treatment, the clinical results and healing process are inferior to those following surgical repairs of small lesions [4, 5].

An alternative by which to achieve better surgical results for massive rotator cuff tears is the use of tissue-engineered tendons, and current research has shown that scaffolds are a reliable alternative that can be used similarly to tissue-engineered tendons [6, 7]. An ideal scaffold for tendon disorders and rotator cuff tears should be (a) three-dimensional in structure with high porosity; (b) devoid of cellular material to minimize the inflammatory potential, disease transmission, and host immune response; (c) cytocompatible; and (d) of sufficient biomechanical capacity to withstand rehabilitation until complete remodelling has occurred [8, 9].

Scaffolds are classified as synthetic or biological. Synthetic scaffolds are generally polymers that may permit satisfactory tissue integration and facilitate cell growth. In orthopaedic practice, poly-urethane urea (SportMesh®, Arthrotek, Warsaw, IN, USA), poly-L-lactide (X-repair®, Synthasome, Del Mar, CA, USA) and polytetrafluoroethylene (Gore-Tex®, Gore, Flagstaff, AZ, USA) have been approved by the Food and Drug Administration (FDA) and have been used in the United States of America (USA) [10, 11].

In contrast, biological scaffolds are composed of complex biological materials such as extracellular matrix (ECM) and cellular remnants [12]. Biological scaffolds are classified as follows according to the origin: autografts (the donor and recipient are the same subject), allografts (the donor and recipient are different subjects of the same species) and xenografts (the donor and recipient are subjects of different species) [13]. Autografts are most commonly used in orthopaedic surgeries; however, their limited availability is prompting studies to develop new scaffolds [13]. Allografts are an available option, but the potential chronic immune reaction and the potential risk of disease transmission are limitations of this scaffold type [14]. Finally, animal-derived xenografts have been associated with immune reactions, resulting in inflammation [14].

One approach to eliminating the risk of disease transmission or immune reactions and improving the availability of scaffolds is the decellularization process, which transforms a scaffold into a shelf product [14–16]. Decellularized tendon scaffolds (DTSs) are prepared from different protocols that include a combination of physical, chemical and enzymatic techniques [17, 18]. Recent studies have shown reproducible results in biomechanical and histological evaluations for protocols that include combinations of chemical and enzymatic decellularization agents [16, 19, 20].

In the literature, grafting in rotator cuff repair has been described, but the results have been mixed [21]. In a recent systematic review, lower re-tear rates and higher scores were found for patients who received rotator cuff repairs plus scaffolds [22]. Regarding the types of grafts used, the poorest results were found for xenografts, and the author did not find significant differences among autografts, allografts and synthetic scaffolds [22]. Several years ago, a randomized clinical trial was terminated due to the development of several local inflammatory reactions in patients treated with rotator cuff repairs plus xenografts (Restore Orthobiologic Implant, Depuy Orthopedics, Warsaw, Indiana) [23].

In this study, we hypothesized that a decellularization protocol with a combination of chemical agents (sodium dodecyl sulfate (SDS) plus t-octyl-phenoxypolyethoxyethanol (Triton X-100)) and enzymatic agents (ethylenediamine tetraacetic acid –(EDTA) plus aprotinin) would be able to produce DTSs with appropriate biomechanical characteristics that could be used in allograft transplantation. This paper aimed to produce and evaluate DTSs from biomechanical, microscopic, macroscopic and in vivo perspectives.

## Methods

### Animal grouping

**Fig. 1:**
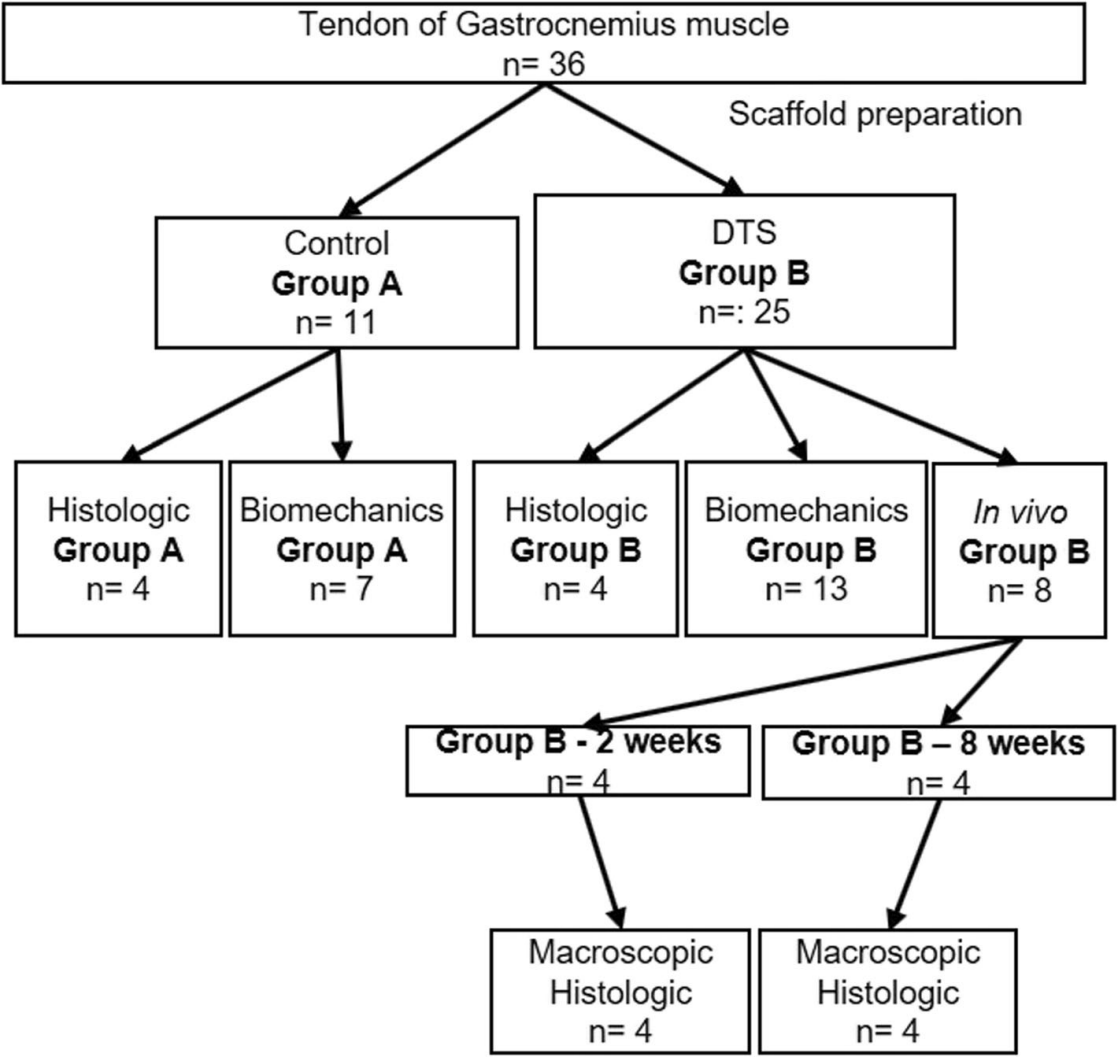
Experimental flowchart detailing the processing and analysis of the control and scaffold

Bilateral gastrocnemius muscle tendons were collected from 18 male New Zealand rabbits between 28 and 32 weeks old and weighing 3–3.5 kg. These rabbits were provided by a farm company (*Granja R. G*., Suzano, SP, Brazil). Of these 36 tendons, 11 were used as controls (Group A - control), and 25 were used in the decellularization protocol (Group B - DTS). Among the 11 tendons used as controls, 4 were subjected to histological evaluation, and 7 were subjected to biomechanical evaluation. Of the 25 tendons subjected to the decellularization protocol, 4 underwent histological evaluation, 13 underwent biomechanical evaluation, and 8 were used for the additional in vivo analysis (Fig. 1). We used 8 (eight) extra rabbits for the in vivo analysis. The study was approved by the Institutional Review Board, and experiments were carried out according to the Animal Research: Reporting of in Vivo Experiments guidelines (ARRIVE).

### Gastrocnemius tendon harvest

Tendons were collected from the rabbits following the administration of anaesthesia and euthanasia with an anaesthetic overdose (ketamine 200 mg/kg + xylazine 40 mg/kg and tramadol 10 mg/kg). Before anaesthesia and euthanasia, the animals were kept in individual cages under a light-dark cycle of 12/12 h with food and water available ad libitum. This study was conducted in accordance with the recommendations for the care and use of laboratory animals.

The leg was cleaned and disinfected using aseptic techniques. With an aseptic technique, a longitudinal medial posterior skin incision was made directly over the flexor digitorum superficialis [24]. The gastrocnemius tendon was carefully dissected from the flexor digitorum superficialis and soleus tendons. A complete transverse laceration was made with a surgical blade through the distal bone insertion and proximal to the muscle-tendon transition. All the tendons were removed and stored at 4 °C in phosphate-buffered saline (PBS) with 1% penicillin/streptomycin (Sigma-Aldrich, St Louis, MO, USA) for the decellularization protocol and biomechanical evaluation of the control or with 10% formaldehyde for the histological evaluation of the control.

### Preparation of DTSs

Immediately after harvesting, the gastrocnemius tendons were transferred under aseptic conditions to individual tubes. Our decellularization protocol had a duration of 6 days, with daily changes of the decellularization agents under aseptic conditions. During the decellularization protocol, an individual tube was placed on an orbital shaker (MaxQ4000, Thermo Scientific, Waltham, MA, USA) at 200 rpm and room temperature.

**Fig. 2:**
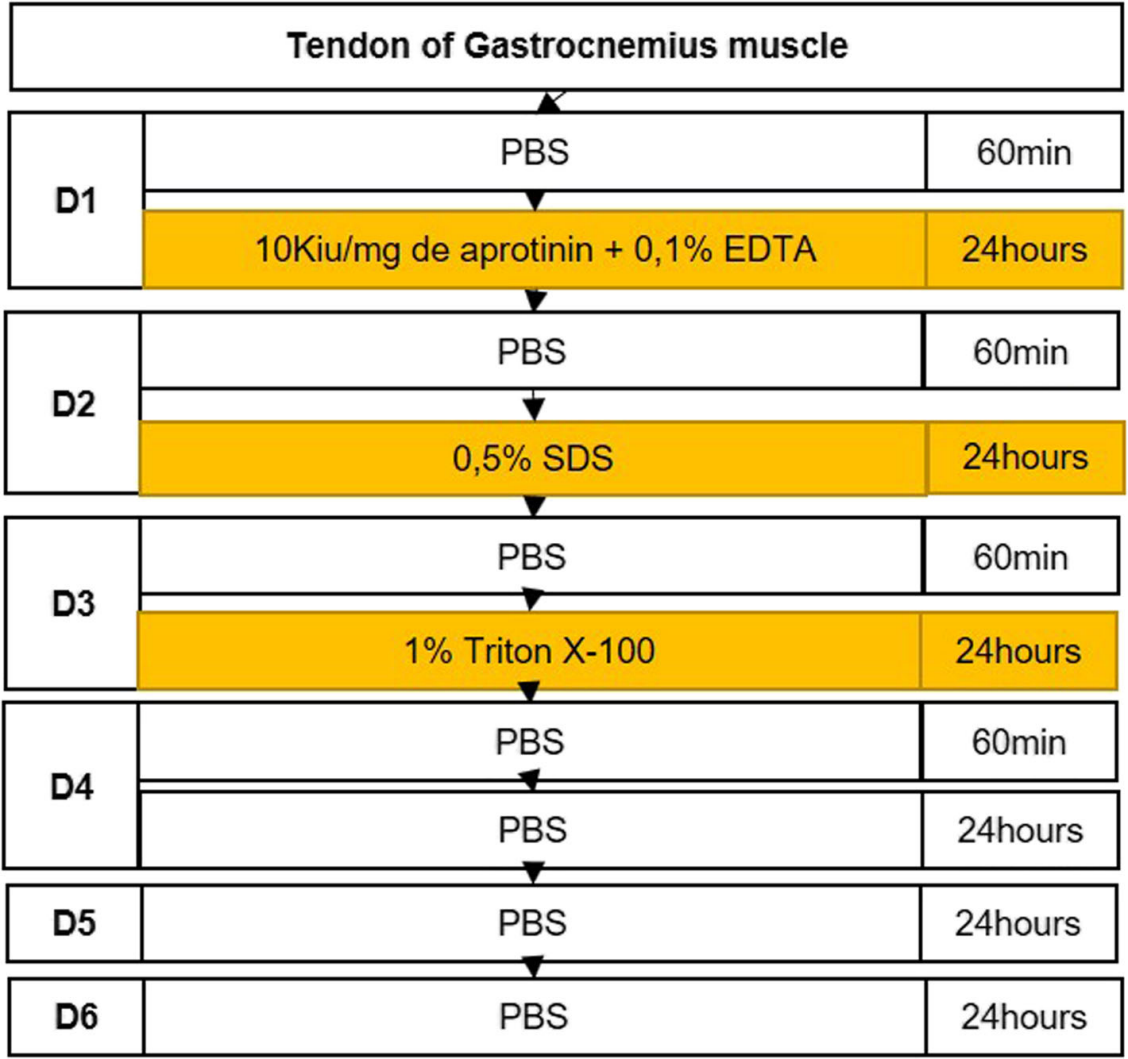
Detailed protocol to produce decellularized tendon scaffolds (DTS). min: minutes, PBS: phosphate-buffered saline, SDS: Sodium Dodecyl Sulfate, Triton X-100: t-octyl-phenoxypolyethoxyethanol

**Fig. 3:**
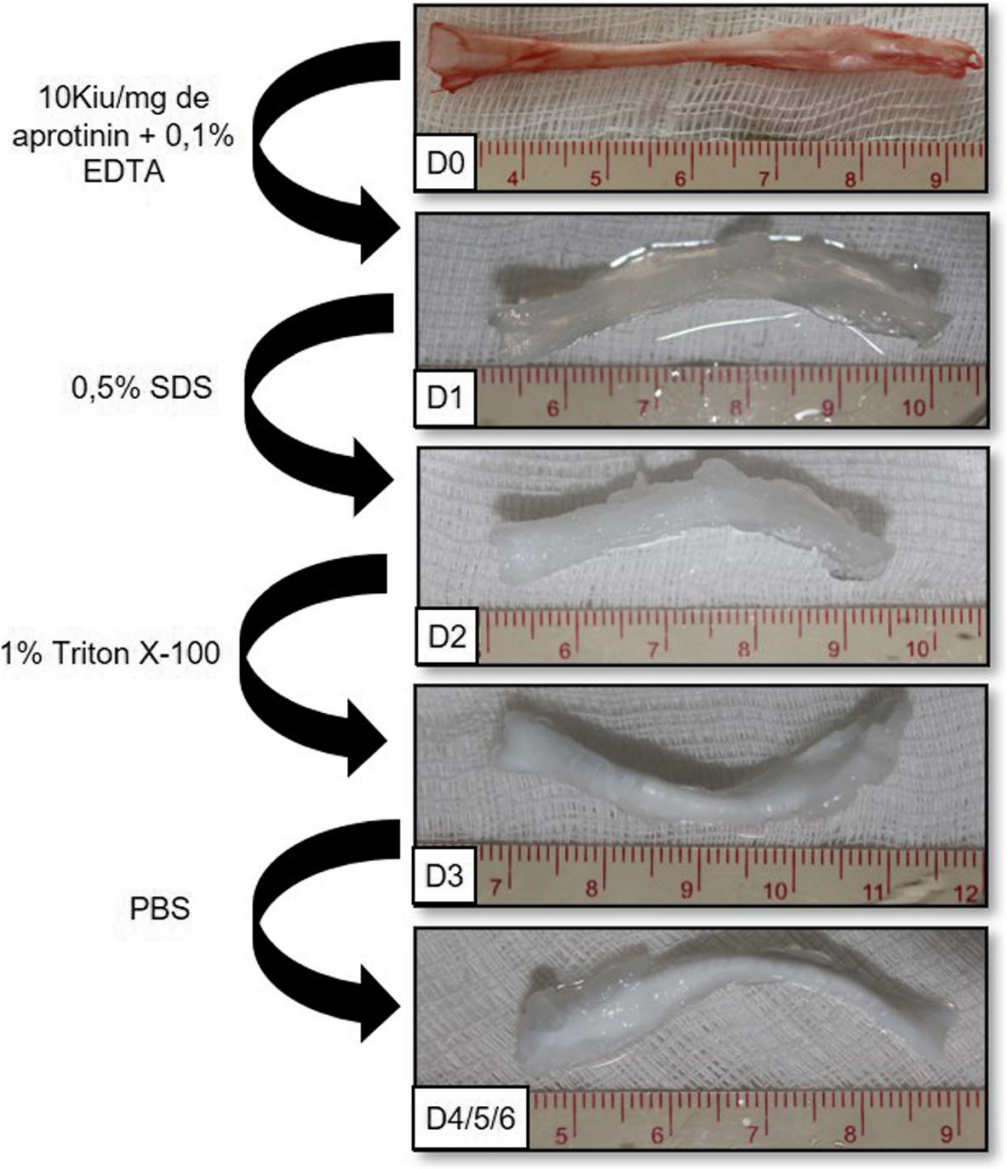
Macroscopic appearance and modifications during the decellularization protocol. Macroscopic morphology has changed to white color and presented the “swelling effect”

In the first step of the decellularization treatment, the tendons were washed with PBS for 60 min and then incubated in a solution containing 0.1% EDTA (Sigma-Aldrich, St. Louis, MO, EUA) and aprotinin 10 K IU/mg (Sigma-Aldrich, St Louis, MO, EUA) for the next 24 h (Fig. 2 and Fig. 3).

Following the decellularization protocol, the tendons were washed with PBS for 60 min and then incubated with 0.5% SDS (Invitrogen, Carlsbad, CA, USA) for 24 h. On the third day, the washing procedure was repeated with PBS, and the tendons were incubated in a solution of 1% Triton X-100 (Affymetrix, Maumme, OH, USA) for 24 h (Fig. 2 and Fig. 3).

On the fourth and fifth days of the protocol, the material was washed daily and maintained under constant agitation in PBS with 1% penicillin/streptomycin (Sigma-Aldrich, St Louis, MO, EUA). On the sixth day, the DTSs were placed in PBS + 1% penicillin/streptomycin (Sigma-Aldrich, St Louis, MO, EUA) and stored at 4 °C.

### Tensile testing of DTSs and gastrocnemius tendons (control)

The DTSs from Group B (*n* = 13) and the controls from Group A (*n* = 7) were removed from storage, and the lengths and cross-sectional areas were measured. The cross-sectional area was measured in the middle of the scaffold or the control with a mini Vernier calliper.

**Fig. 4:**
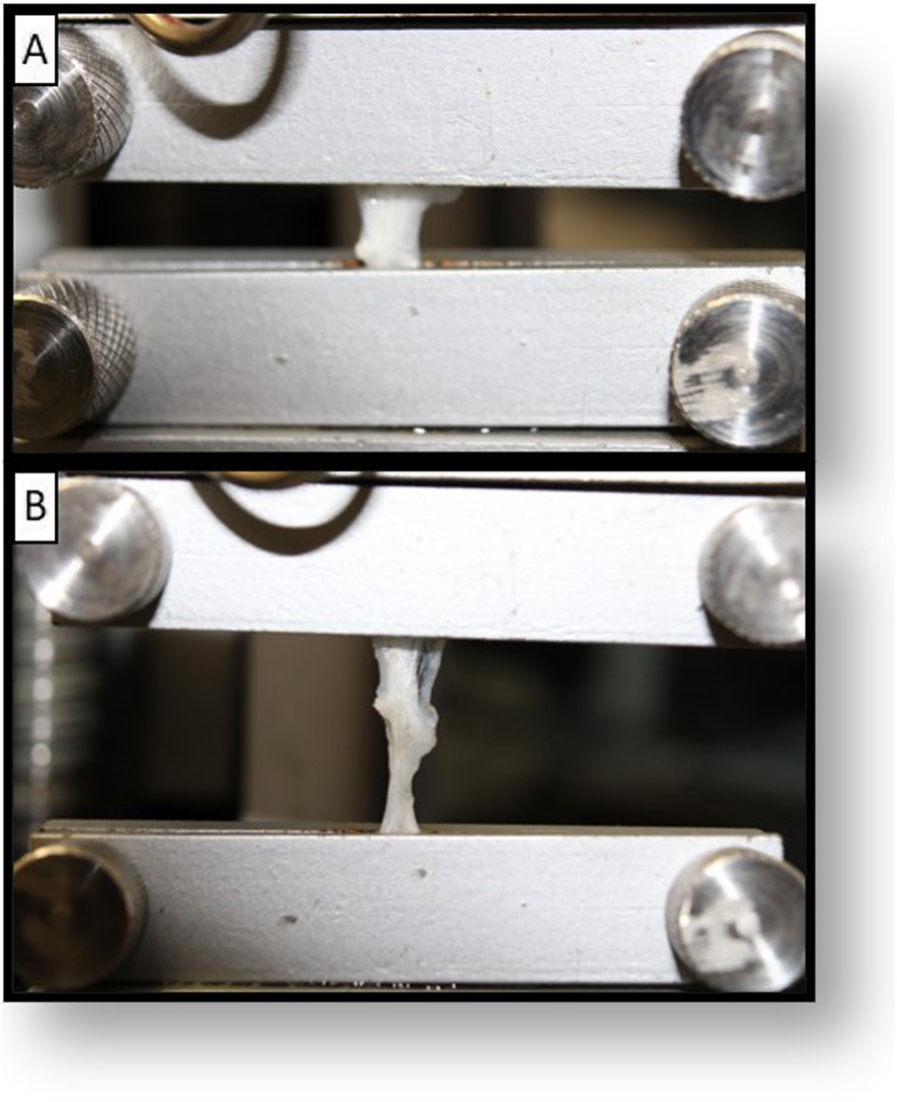
Tensile testing model. **a** Specimen positioning in the customized jig system. **b** Tensile traction procedure showing the progressive specimen rupture

The DTSs or the controls were fixed with a customized jig system consisting of metal cages with adjustable pins (Fig. 4). A specimen was mounted onto a machine for testing (AME equipment - 2KN, Oswaldo Filizola, São Paulo, SP, Brazil) and preloaded at a predefined distance of 5 mm to pre-stretch the material. The specimen was then loaded to failure at a constant speed of 10 mm/min until complete rupture. Immediately after preloading, the tensile load and elongation were recorded using the DynaView Standard M Software *M* (Software DynaView Standard M versão 2.7.5, Técnica Industrial Oswaldo Filizola, São Paulo, SP, Brasil) [25]. We calculated the elastic modulus, ultimate tensile stress, and stiffness using the data collected with the software and a mini Vernier Calliper [26].

### Histological analysis of DTSs and gastrocnemius tendons (control)

Mid-substance portions of the specimen were placed in 10% phosphate-buffered formalin at room temperature for 6 h. The specimens were embedded in paraffin, and a microtome was used to obtain transverse sections of 3 μm thickness. The sections were mounted on slides and stained using haematoxylin and eosin (H & E), Masson’s trichrome, 4,6-diamidino-2-phenylindole (DAPI, Invitrogen, Carlsbad, CA, EUA) and Sirius Red.

The histological sections were analysed using an Olympus IX 81/ BX51 (Olympus Corporation, Shinjuku-ku, Tokyo, Japan) fluorescence/optical microscope and a Zeiss AX10 (Zeiss, Jena, Germany) optical microscope, and images were captured with an Olympus DP71/DP72 (Olympus Corporation, Shinjuku-ku, Tokyo, Japan) or Zeiss AxioCam ICc5 (Zeiss, Jena, Germany) camera. The histological evaluation was based on the presence and organization of nuclear components, as well as the organization of the scaffold architecture [8, 20]. Nuclear material was evaluated in a descriptive analysis using H&E and DAPI-stained samples. We classified nuclear basophilia removal as complete, substantial, or no removal [27]. The architectural preservation was evaluated in a descriptive analysis using H&E, Masson’s trichrome and Sirius Red-stained samples [8]. We graded the architecture modification as normal, minimal disruption, or marked disruption.

### Macroscopic analysis of DTSs and gastrocnemius tendons (control)

Macroscopic analysis was performed on all DTSs and controls. We analysed differences in colour, appearance, and size post-decellularization. All material collected was documented with photographs, and the results were evaluated using photos and direct visualization. The gross morphology was evaluated by two blinded participants (LSSB and CGS). Any disagreements were resolved by involving a third author (FGA).

### In vivo host inflammatory response and tissue integration of DTSs in a rotator cuff model

For the in vivo evaluation, we used 8 additional male New Zealand rabbits between 28 and 32 weeks old and weighing 3–3.5 kg. These rabbits were provided by a farm company (*Granja R. G*.). We divided the animals into two groups. Those in “Group B – 2 weeks” were euthanized after 2 weeks, and those in “Group B – 8 weeks” were euthanized after 8 weeks (Fig. 1). For this evaluation, we created lesions in the bilateral shoulders, but we placed a DTS only on one side, and the contralateral side served as the control. The animals were anaesthetized with a combination of ketamine 50 mg/kg + xylazine 10 mg/kg + tramadol 10 mg/kg, and a combination of tramadol 10 mg/kg and meloxicam 2–3 mg/kg was used for analgesia. The animals were kept in individual cages under a light-dark cycle of 12/12 h with food and water available ad libitum. The animals were not maintained with postoperative cast immobilization.

#### In vivo host cell infiltration and inflammatory response to DTSs in a rotator cuff tear model

**Fig. 5:**
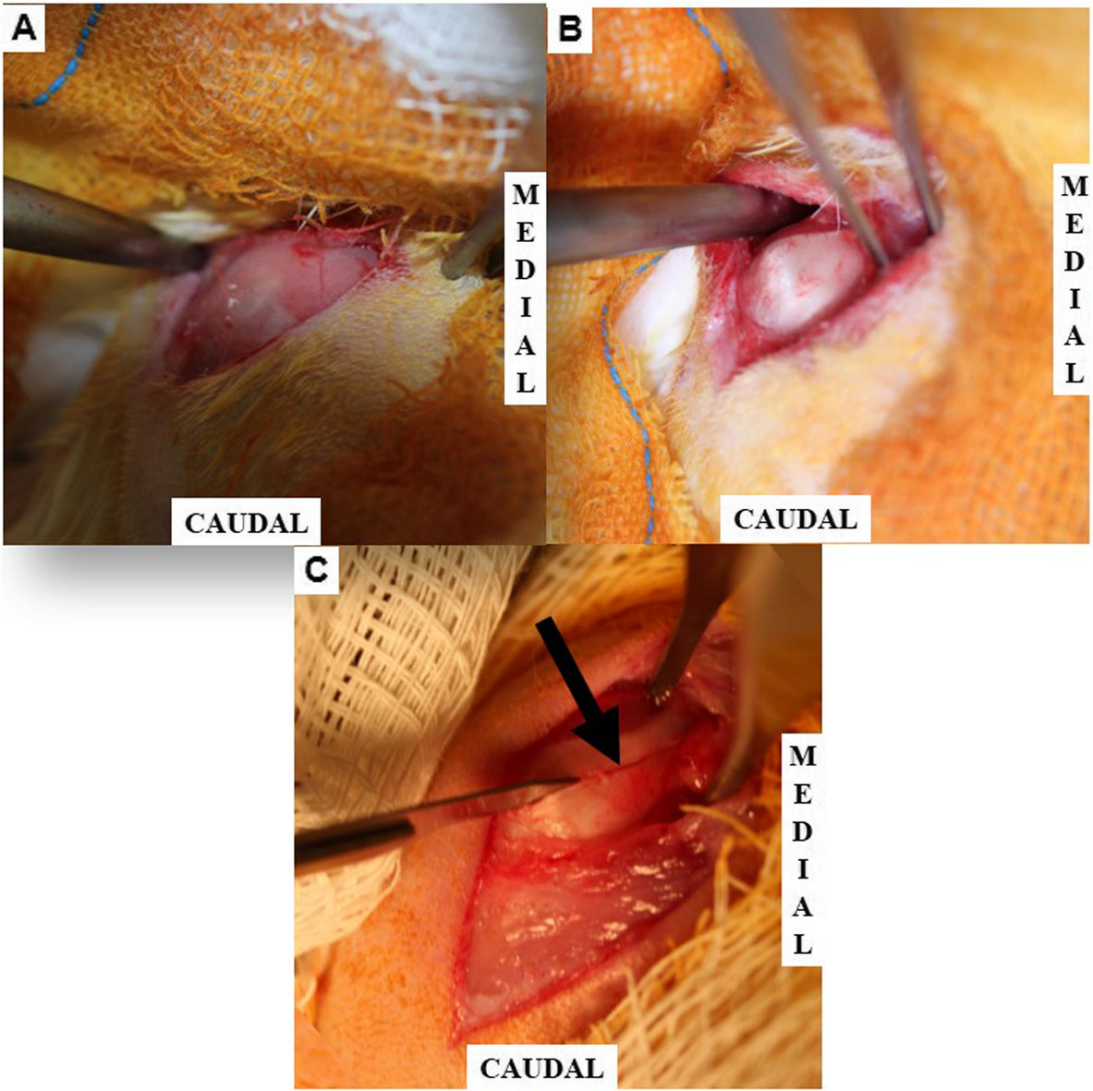
The experimental model of rotator cuff lesion. **a** Exposition of the deltoid muscle. **b** Exposition of the rotator cuff (subscapularis tendon). **c** Rotator cuff lesion without detachment. Arrow: Lesion of the rotator cuff tendon

**Fig. 6:**
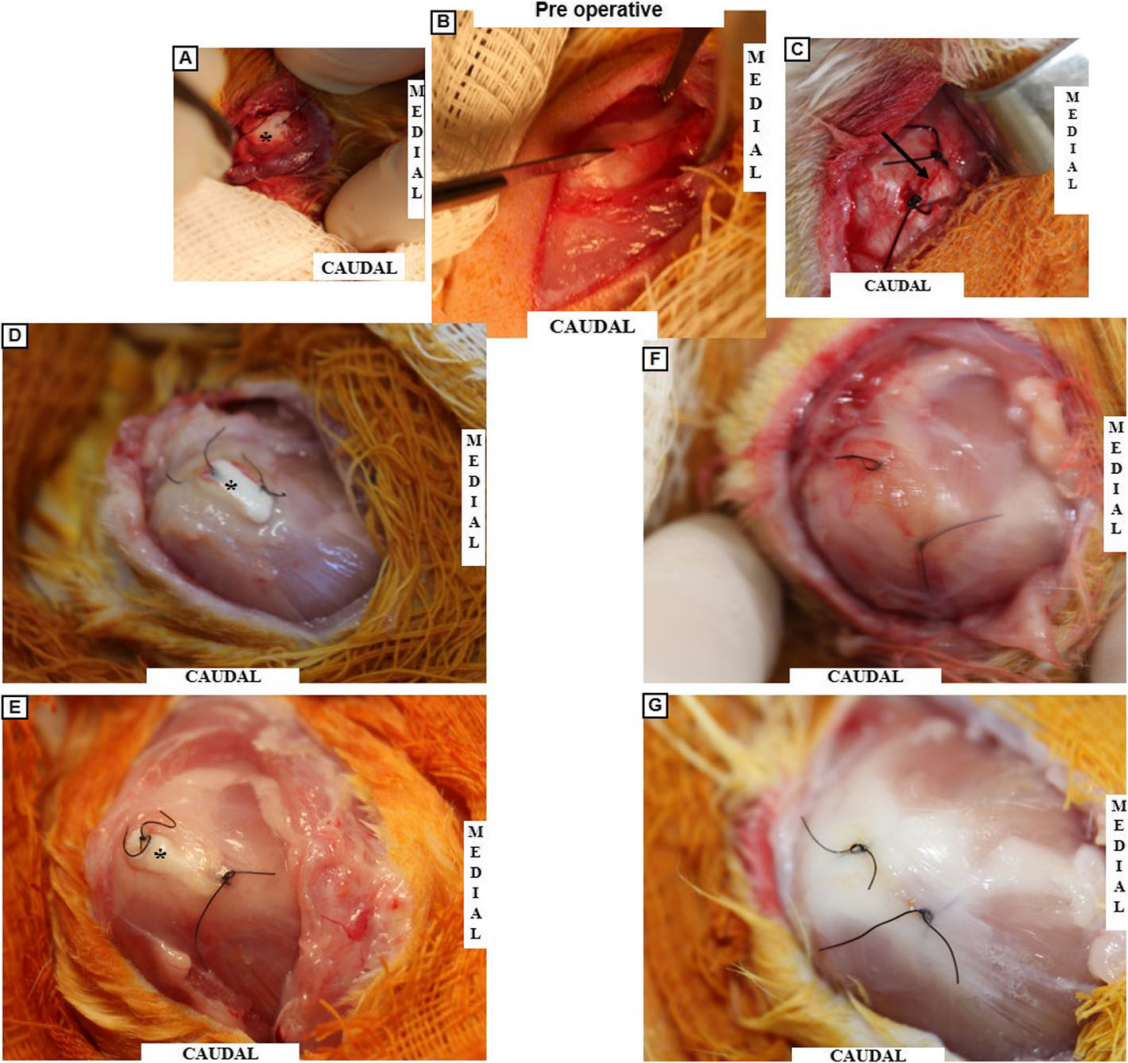
Macroscopic In Vivo *e*valuation. **a-c** Pre-operative characteristic. **d-e** Group B – 2 and 8 weeks post-operative. **f-g** Control Group B – 2 and 8 weeks post-operative. **a** Pre-operative visualization showing the DTS positioned in the rotator cuff lesion. **b** Rotator cuff lesion experimental model. **c** Pre-operative visualization showing the control. **d** 2 weeks of postoperative DTS insertion, showing inflammatory reaction and no signal of rejection. **e** 8 weeks post-operative appearance showing tissue integration between the DTS/tendon and a more organized tissue. **f** Control appearance after 2 weeks showing inflammatory tissue in the region of the lesion. **g** Control appearance after 8 weeks showing a cicatrized tendon. (*) Asterisk representing the DTS topography. Arrow: indicating the lesion region, DTS: decellularized tendon scaffold

Following the approved anaesthetic protocol, the bilateral subscapularis tendons of each animal were injured using an aseptic technique [28]. A anterolateral incision was made on the shoulder (Fig. 5-a), and the subscapularis was exposed after deltoid splitting (Fig. 5-b). A complete parallel laceration without detachment was made in the bilateral subscapularis tendons (Fig. 5-c). The laceration was not repaired, and a DTS was placed on only one side (Fig. 6-a). The contralateral side was used as the control (Fig. 6-c). We used Nylon 4/0 (Nylon 4–0, Shalon, Alto da Boa Vista, GO, Brazil) to mark the scaffold and the lesion on the contralateral side. The animals used for the in vivo evaluation were subdivided into two groups and euthanized with an anaesthetic overdose (ketamine 200 mg/kg + xylazine 40 mg/kg and tramadol 10 mg/kg) after two or eight weeks. The subscapularis tendon was resected from the musculotendinous junction to the bone insertion.

A histological evaluation was performed on the scaffold topography, and we evaluated the differences between specimens at two and eight weeks in terms of the cell infiltration, inflammatory response, and collagen arrangement. In the second step, we evaluated the gross morphology of the integration between the DTS and tendon and the inflammatory reaction [7, 8].

### Statistical analysis and sample size calculation

The number of animals used for the biomechanical evaluation was determined a priori by a significance level of 0.05 and statistical power of 95% using the mean difference plus standard deviation (SD) between the ultimate tensile load of a DTS (198.24 ± 21.05) fabricated by a similar protocol and the control (253.78 ± 12.36) [20]. Using this methodology, the minimum number of samples per group was 5 (five). In a second approach, we compared the number of samples with those in relevant literature [8, 20].

Statistical analyses of the biomechanical data were performed by a nonparametric test (the Wilcoxon-Mann-Whitney test) and a power *post-hoc* test (Welch-Satterthwaite two sample t-test). *P* ≤ 0.05 was set as the level of statistical significance. Statistical data analysis was performed using the *software* SAS® Studio (SAS 3.8 Basic Edition; SAS Institute, Cary, NC, USA) and Excel® (Microsoft® Excel® para Office 365 MSO; Santa Rosa, California, USA).

## Results

### Tensile testing of DTSs and gastrocnemius tendon (control)

During tensile traction, all the specimens showed a rupture in the space between the customized jig system (Fig. 4). If the specimens slipped out of the jig system, the result was discarded. In this study, we did not observe slippage during tensile testing.

**Fig. 7:**
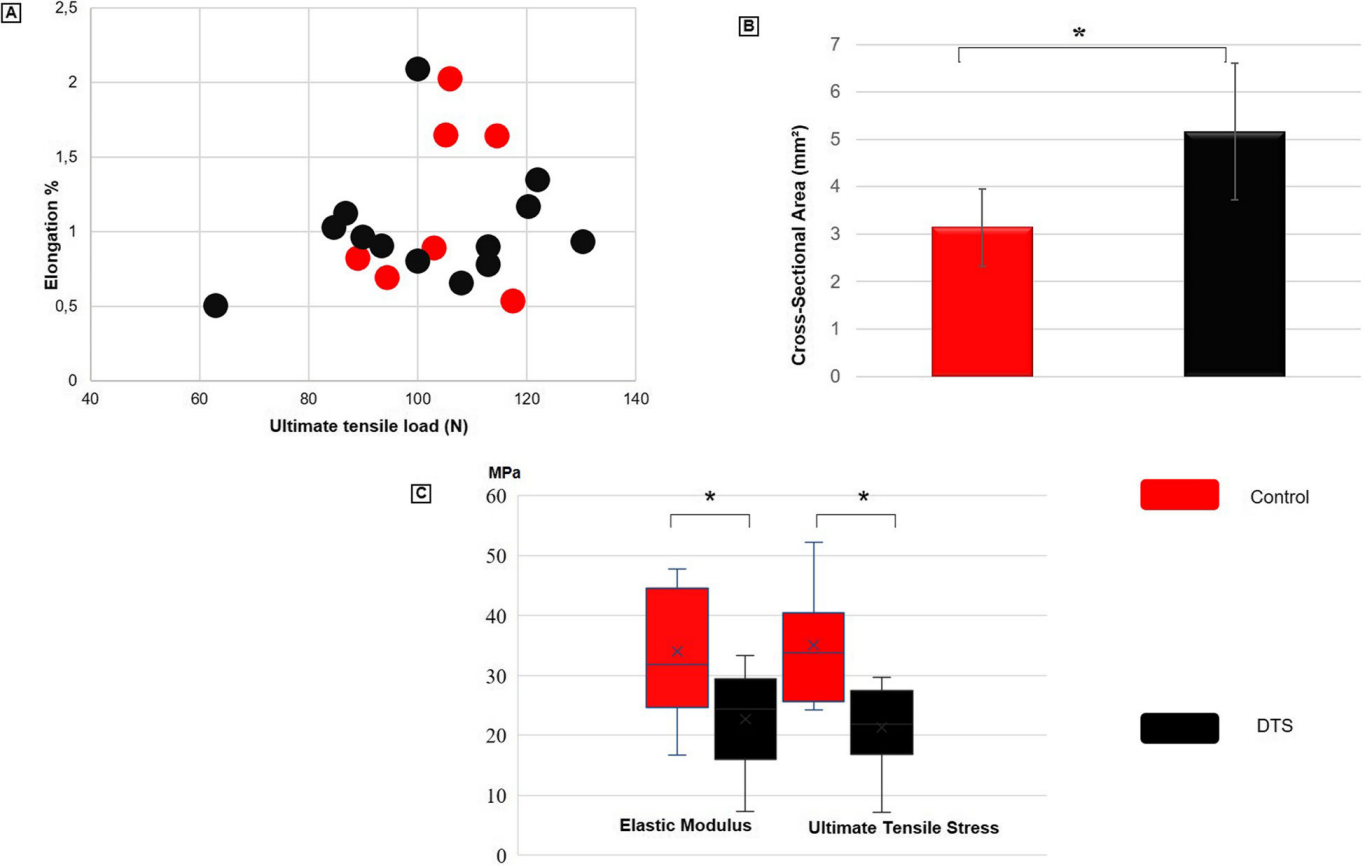
Biomechanical property testing with control and DTS. **a** Elongation (%) X Ultimate tensile load (N) in a dot plot presentation. **b** Graphic representation of mean values for cross-sectional area (mm^2^). **c** Graphic representation of mean values for ultimate tensile strain (MPa) and elastic modulus (MPa). (* *p* < 0.05). mm^2^: Square millimeter, MPa: Megapascal, N: Newton, DTS: decellularized tendon scaffold

The ultimate tensile load, stiffness, and elongation at the ultimate tensile load did not significantly differ between the groups (Table 1 and Fig. 7). The ultimate tensile load supported by the DTS was approximately 98% of that of the control. The stiffness values of the two groups were similar, and the elongation at the ultimate tensile load of the DTS was approximately 86% of that of the other group (Table 1).

**Table 1:** Comparative table (control x DTS) relative to the biomechanical properties. No significant difference was observed in the failure load among the groups in the data relative to the ultimate tensile load, elongation at the ultimate tensile load, and stiffness. (* *p* < 0.05)

	Group I – Tendon	Group II - DTS	Scaffold / Tendon Ratio	Statistical power
Ultimate tensile load (N), SD	104.18 (10.11)	101.9 (18.46)	98%	0.061
Elastic modulus (MPa), SD *	33.98 (11.52)	22.65 (7.94)	67%	0.596
Elongation at the ultimate tensile load, SD	1.18 (0.58)	1.01 (0.39)	86%	0.099
Stiffiness (N/mm), SD	21.87 (11.45)	21.99 (6.34)	101%	0.053
Cross-sectional-area (mm^2^), SD *	3.14 (0.81)	5.16 (1.44)	164%	0.965
Ultimate tensile stress (MPa), SD *	35.04 (9.68)	21.28 (6.61)	61%	0.844

*p < 0.05

Legend: *N* Newton, *SD* Standard Deviation, *Mpa* Megapascal, *mm* millimeter, *mm*^*2*^ Square millimeter, *DTS* Decellularized tendon scaffold

The cross-sectional area, ultimate tensile stress, and elastic modulus showed statistically significant differences between the groups (Fig. 7). The cross-sectional area of the DTSs increased by 164% compared to those of the controls. The elastic modulus and ultimate tensile stress of the DTSs were reduced by 61% compared to those of ​the control (Table 1). In the *post-hoc* analysis, the cross-sectional area and ultimate tensile stress showed power superior to 80%, and the elastic modulus showed a power superior to 50%. The other variables evaluated showed a power inferior to 50%.

### Histological analysis of DTSs and gastrocnemius tendon (control)

**Fig. 8:**
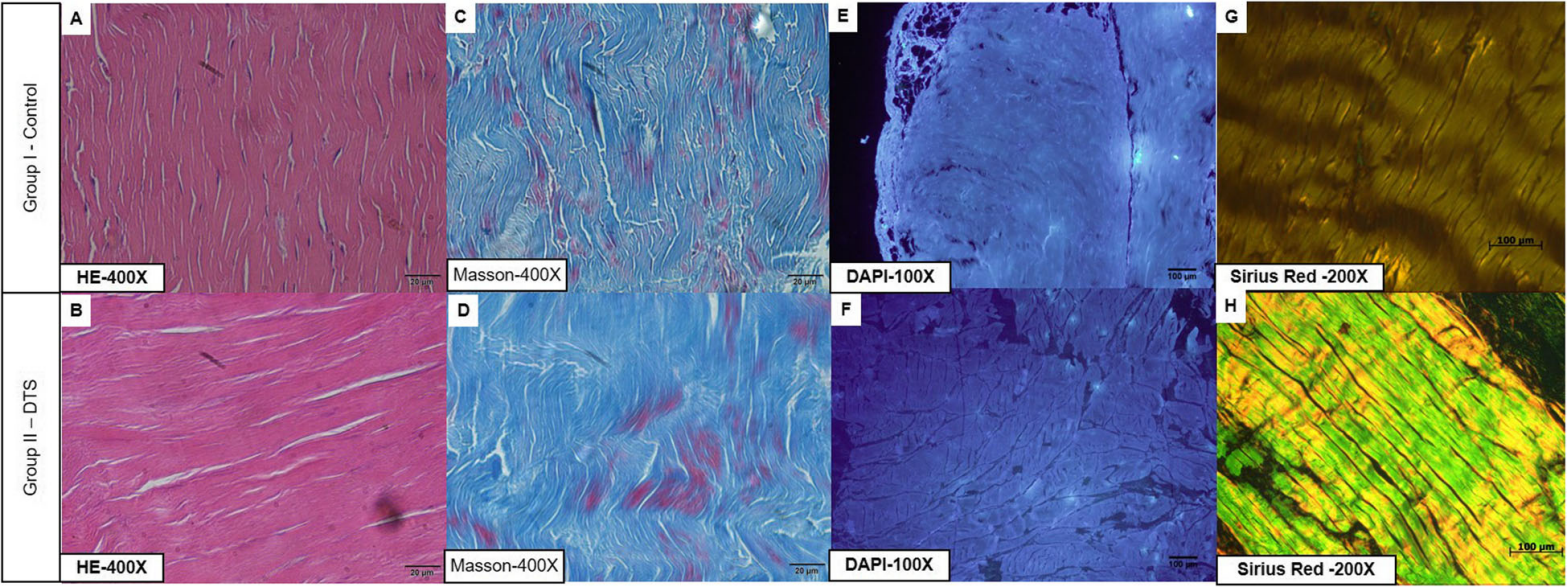
Histological Analysis. **a** Material cellular and tendon architecture were visible. **b** Minimal alterations in the scaffold architecture and substantial nuclear remotion were noted. **c** Representative slides with the normal architecture of the tendon. **d** Trichome Masson slides showing minimal alteration in the architecture structure. **e** Punctuate nuclei (DNA) were visible in the control. **f** Only disrupted and disorganized DNA remains in the scaffold slides. **g** The normal architecture of the control. **h** Representative slides showing minimal disruption of the architecture. DTS: decellularized tendon scaffold

The controls had abundant nuclear material, as observed especially in the H&E and DAPI staining experiments, as well as intense organization of the ECM in the H&E, Sirius Red and Masson’s trichrome staining experiments (Fig. 8).

The DTSs showed no nuclear material in H&E staining, but DAPI staining showed the presence of nuclear material. However, in the DTSs, the nuclear material was not as organized as it was in the controls. In conclusion, we noted a substantial loss of nuclear material (Fig. 8).

We noted increases in the spaces between the collagen structures, particularly in the inter and intrafascicular spaces (Fig. 8). However, the parallelism of the fibres did not change in the DTSs, as observed in the H&E, Masson’s trichome and Sirius Red staining results (Fig. 8). In conclusion, we noted minimal disruption in all the decellularized scaffolds.

### Macroscopic analysis of DTSs and gastrocnemius tendons (control)

The macroscopic analysis revealed an approximately original shape, and no pink colouration (related to eventual remaining vascularization) was observed. The collagen was well aligned, but we noted an increase in the volume of the scaffold (Fig. 3).

### In vivo host cell infiltration and inflammatory response to DTSs in a rotator cuff tear model

#### Histological analysis of in vivo host cell infiltration and inflammatory response to DTSs

**Fig. 9:**
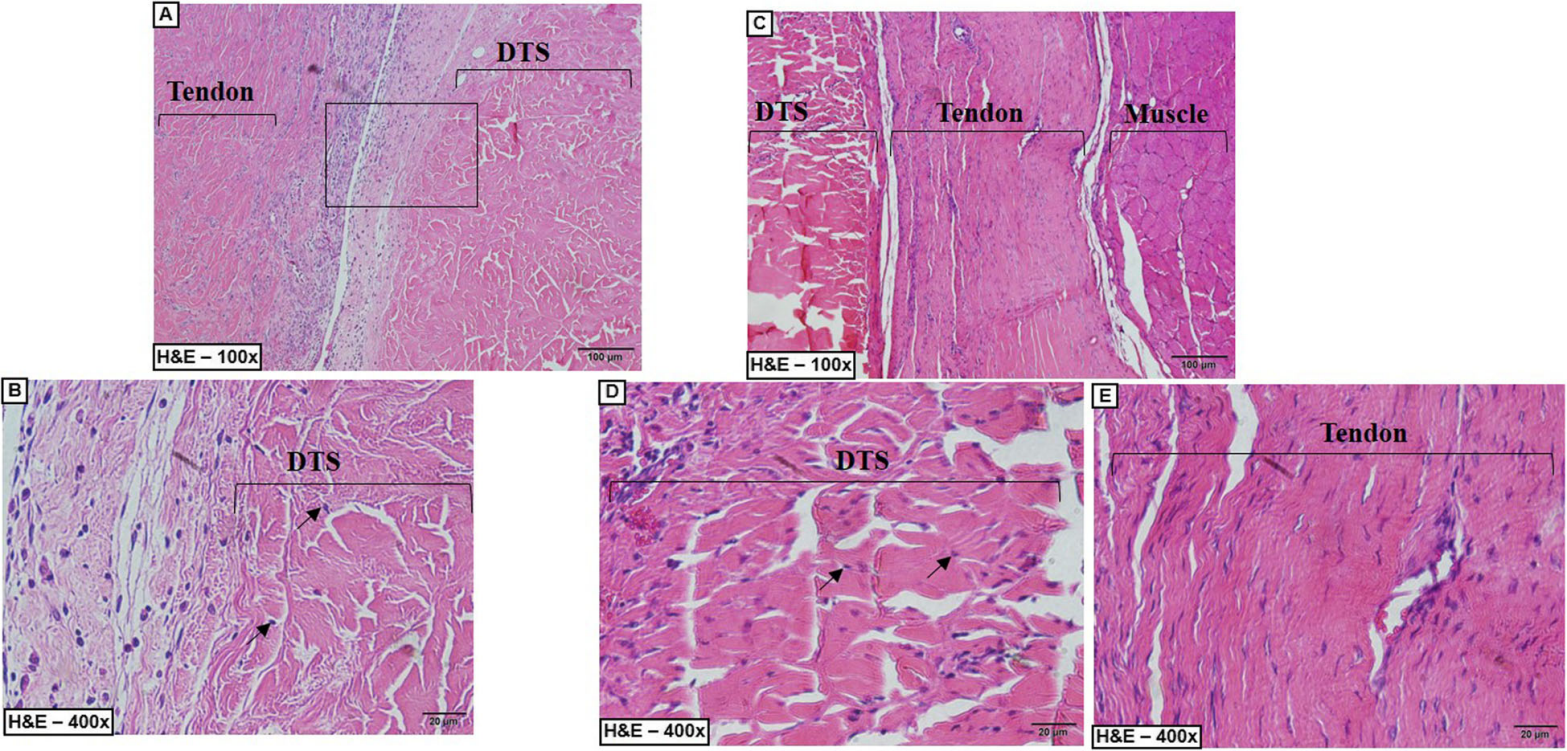
Representative slides of the In vivo evaluation. **a**-**c** Group B – 2 weeks of post-operative. **c-e** Group B – 8 weeks of post-operative. **a** A large number of inflammatory cells between the tendon and the DTS. **b** Mononuclear cell infiltration (arrow) were visible in the periphery of the DTS. **c** Organized tissue showing the rotator cuff after the cicatrization and the integration of the DTS with the native tissue. **d** Mononuclear cell infiltration (arrow) were visible in the periphery and central region of the DTS. **e** Organized native tendon after 8 weeks of post-operative. DTS: decellularized Tendon Scaffold, arrow: cell infiltration

We used H&E-stained sections to evaluate the cell infiltration and inflammatory reaction. Cell infiltration of fibroblast-like host cells was observed in the DTSs, and we found more infiltration after 8 weeks than after 2 weeks (Fig. 9). Cell infiltration was concentrated in the extremities of the DTS after 2 weeks and disseminated in the central region after 8 weeks (Fig. 9).

**Fig. 10:**
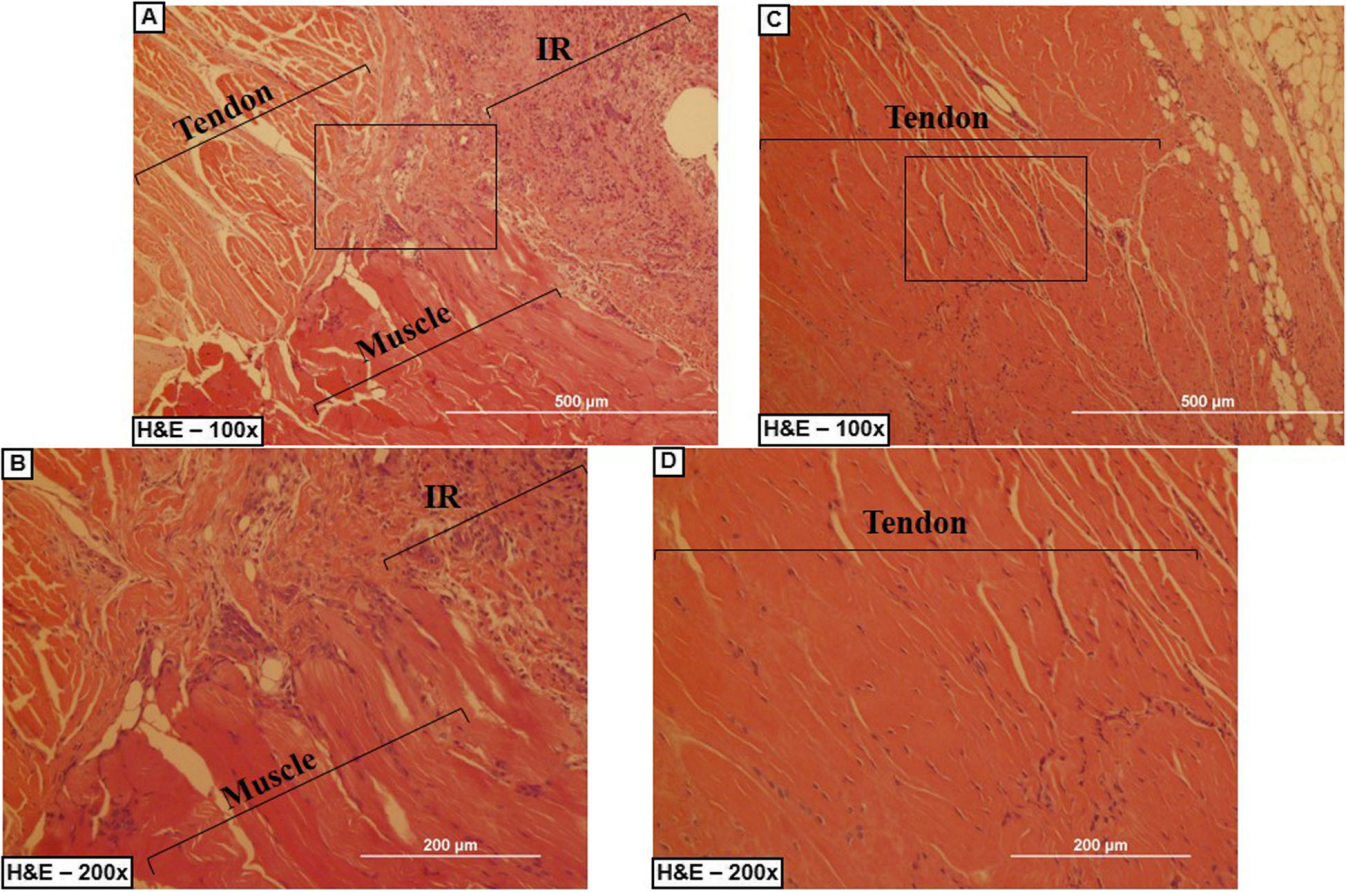
Representative slides of the control at the In vivo evaluation. **a-b** Control Group B – 2 weeks of post-operative. **c-d** Control Group B – 8 weeks. **a** Evident inflammatory reaction (IR), and disorganized tendon/muscle tissue. **b** inflammatory reaction (IR), with a large number of linfocits and macrophages. **c-d** Organized tissue showing the rotator cuff cicatrization. IR: Inflammatory Reaction

In the inflammatory evaluation, we did not find significant evidence of capsule or granuloma formation, but we found some inflammatory cells, such as lymphocytes and macrophages, near the DTS, mainly after 2 weeks (Fig. 9 and Fig. 10).

#### Macroscopic analysis of in vivo host cell infiltration and inflammatory response to DTSs

No signs of weight loss, infection, or wound spliting were observed. In the two groups, the subscapularis insertion was in its anatomical location (Fig. 6), and the interface DTS/rotator cuff could not be detached without damaging the DTS (Fig. 6 d/e). We noted some differences between the two groups. In “Group B – 8 weeks”, the connective tissue between the DTS and the rotator cuff was more abundant and the inflammatory reaction was less evident than in “Group B – 2 weeks” (Fig. 6 d/e).

## Discussion

Our most important findings were that the DTSs had a similar ultimate tensile load to that of a normal tendon, a high porosity, no organized nuclear material and showed tissue integration in the rotator cuff model. Scaffolds generated from decellularization protocols are currently used in clinical practice in various specialties [29, 30]. In developing countries, the high cost and regulatory services limit the use of these scaffolds, unlike in developed countries. Our purpose was to fill that gap and to reproduce the promising results obtained in other studies [8, 31]. We thus evaluated a decellularization protocol with high reproducibility using low-cost technology. We consider two options for the future. First, we could use rabbit-derived xenografts, or we could use this protocol to prepare decellularized human tendons for use in allograft transplantation. Currently, we believe that decellularized allograft transplantation is a viable option with a low likelihood of rejection.

Regarding the decellularization process, an efficient protocol should include a combination of chemical (detergents), biological (enzymatic or non-enzymatic agents) and physical agents [19, 32]. This protocol must be applied for extended periods with an appropriate technique, depending on the materials to be decellularized [17, 32]. Our protocol used a combination of two detergents (SDS + Triton X-100), a non-enzymatic agent (EDTA), a protective compound for the ECM (aprotinin), and PBS to wash the DTSs. Furthermore, the DTSs were maintained under constant agitation, following the recommendations for tendinous structures [17, 32].

The histological analysis showed an acellular scaffold, which may be related to the reduced immunogenicity and antigenicity and may maximize the host cell infiltration into the scaffold in vivo or in vitro. The biomechanical analysis showed a statistically significant increase in the cross-sectional area and decreases in the ultimate tensile stress and elastic modulus. These alterations are related to the swelling effect and did not change the minimal requirements for human tendon repair [33]. Based on analysing the biomechanical alterations individually, the increase in the cross-sectional area may be related to the high porosity, which may facilitate host-cell infiltration [34]. Furthermore, the elastic modulus is a direct measure of the amount of energy absorbed by the tendon, and a higher elastic modulus may be related to a stiffer and smaller deformation of the biomaterial in physiological situations [35].

Despite the biomechanical alterations related to the swelling effect, the ultimate tensile load, the elongation at the ultimate tensile load and the stiffness did not change with the protocol. In our opinion, if the ultimate tensile load and elongation are not different from those of a normal tendon, the scaffold can be used as a tendon substitute and facilitate early patient rehabilitation.

We noted some limitations. First, we did not measure the immunogenicity, toxicity, or antigenicity, and we do not know whether these biomechanical properties will persist after the use of such DTSs as a graft. The toxicity related to the decellularization process was not directly evaluated in this study, and in the clinical use of the DTS, this could be a difficulty to be solved. Second, no consensus is available on the best protocol for sterilization, which is one of the difficulties of DTSs. We attempted to use an ethylene oxide protocol as a pilot test, but the results were not satisfactory. One solution to this problem is to use peracetic acid in the protocol as an additional sterilization protocol [8, 31, 36].

An important additional limitation is related to the a priori sample size estimate. After the *post-hoc* analysis, we have noted that half of the biomechanical results had a statistical power inferior to 50%, and the results could be underpowered.

Some innovations related to this work should be noted. This is a new approach to using DTSs in orthopaedic procedures in a country with a large lack of soft tissue scaffolds, and we are the first to report using DTSs in a rotator cuff tear model.

In the future, we hope to start testing scaffolds in several orthopaedic procedures. DTS application in a massive rotator cuff lesion model and for anterior cruciate ligament reconstruction will be our next objectives. A different method for these studies may involve the use of mesenchymal stem cells (MSCs) as an adjuvant in the same orthopaedic procedures [25]. For these subsequent studies, the final objective will be to realise the off-the-shelf availability of reliable DTSs for use in clinical practice.

## Conclusion

The evaluated decellularization protocol generated a tendon scaffold, maintained the most important biomechanical characteristics and permitted cell infiltration.

## Data Availability

The datasets used and/or analyzed during the current study are available from the corresponding author on reasonable request.

## Abbreviations

DAPI: 4,6-diamidino-2-phenylindole
DTS: Decellularized Tendon Scaffolds
EDTA: Ethylenediamine tetraacetic acid, 0,1% w/v
FDA: Food and Drug Administration
H & E: Haematoxylin and eosin
IR: Inflammatory Reaction
min: Minutes
mm: Millimetre
MPa: Megapascal
MSC: Mesenchymal stem cell
N: Newton
PBS: Phosphate-buffered saline
rpm: Rotations por minute
SD: Standard deviation
SDS: Sodium Dodecyl Sulfate
Triton X-100: T-octyl-phenoxypolyethoxyethanol
TnBP: 1% tri-n-butyl phosphate
USA: United States of America
